# Steps Toward Justice: a model for equitable involvement of young people in mental health promotion

**DOI:** 10.3389/fpubh.2025.1636799

**Published:** 2025-11-13

**Authors:** Helena Gard, Gabriella Elisabeth Isma, Maria Petersson, Helene Sjöblom Andersson, Elisabeth Mangrio, Karin Enskär, Karin Ingvarsdotter

**Affiliations:** 1 Department of Care Science, Malmö University, Malmö, Sweden; 2 Culture and Leisure, Administration of the Municipal Board, Municipality of Karlshamn, Karlshamn, Sweden; 3 Blekinge Center for Competence, Regional Council of Blekinge, Karlskrona, Sweden; 4 Department of Women’s and Children’s Health, Uppsala University, Uppsala, Sweden; 5 Department of Social Work, Malmö University, Malmö, Sweden

**Keywords:** health promotion practices, participatory action research, youth involvement, youth mental health promotion, youth participation

## Abstract

**Introduction:**

Mental ill-health affects young people being marginalized to a greater extent than other young people. Nevertheless, are groups being marginalized underrepresented in health research and practice. Several models regarding youth participation have been developed, but knowledge is still lacking on how health promotion practitioners can equitably involve young people when developing health promotion efforts.

**Aim:**

This study aims to (1) describe how participatory action research was used to develop a model for practitioners to equitably involve young people in mental health promotion initiatives and (2) present the finalized model, Steps Towards Justice.

**Methods:**

Through a participatory action research approach, a group of practitioners constructed a model for an equitable involvement of young people in mental health promotion. The model was developed further together with focus groups of practitioners and young people.

**Results:**

The finalized model consists of three phases: preparing, conducting, and applying, with different steps of action to be taken in each phase. To identify inclusion and exclusion, practitioners need to be critical and aware of their own prejudice and values throughout the process. The practice of involving young people equitably also includes creating safe spaces and valuing young people of different backgrounds equally in their contribution to mental health promotion.

**Conclusion:**

The model can be useful for practitioners wanting to involve young people when planning and conducting mental health promotion, for instance at schools or youth centers. It can foster the process of critical reflection around equitable practices and taking steps toward justice through concrete actions of involvement, moving beyond a vague discourse of “everyone is welcome.”

## 1 Introduction

Being marginalized, regardless of age, negatively affects mental health. Accordingly, youth mental health varies between different groups of young people in the population. Research has shown that processes related to socioeconomic status, gender, family structure, and country of birth seems to contribute to youth mental health inequities in the Nordic countries (1). Further, young people experience inequities and injustices in their everyday life, as race, gender, and socioeconomic status relate to challenges young people face in their everyday life (2). As these injustices are experienced in everyday life, they must also be considered in youth mental health promotion. For example, socioeconomic status seems be related to which changes young people see as important for reducing stress and coping with everyday challenges, and also to how young people wanted to participate themselves in making these changes (3).

Mental health experts, both professionals and young people, problematize underrepresentation of certain groups of young people in youth mental health research. In particular, young people with migration experience, young people with disabilities, and young people from low socioeconomic status settings were considered underrepresented in all forms of youth involvement in mental health research (4). Groups and individuals often underrepresented in research are sometimes referred to as “hard-to-reach” or “hidden group” and different methods are used to try to reach these groups (5). This terminology has, however, also been criticized as it enforces othering and places the problem on groups being marginalized, instead of addressing the responsibility of the health researcher to conduct inclusive and participatory research (6). Children being minoritized are also generally underrepresented in health and medical research, possible explanations could be logistic barriers such as transportation and language as well as mistrust in research institutions due to previous experiences of discrimination (7).

Theoretical perspectives informing child and youth participation include children’s rights, an individual perspective, and childhood sociology, considering also structural and relational perspectives, viewing childhood as socially constructed (8, 9). Through the different theoretical perspectives, several different approaches for youth participation are used in public health research, policy development, and health promotion (10, 11). Commonly used models for youth participation include the Ladder of Participation by Hart (12) and the Pathways to Participation by Shier (13); these models are often used to plan, conceptualize, and evaluate youth participation from a children’s rights perspective. Sweden ratified the United Nations Convention of the Rights of the Child in 1990, and adopted it as Swedish legislation in 2020. Adopting the convention as legislation aimed to strengthen the rights of children in all decision-making processes that concern children, to strengthen child participation, and to develop a child-oriented approach in the public sector (14). This has increased awareness among public organizations and practitioners about the importance to include children themselves when developing efforts aimed at children, but the impact of adopting the convention as legislation remains unclear (15).

Within public health research and health promotion, specific motivations for involving young people seem to be both to benefit the health outcome by having young people contribute with their knowledge and expertise to create relevant and sustainable actions, but also to empower and strengthen the individual young people who participate (11). Involving young people in developing public health policy seems to have a positive effect on the personal development of the young people participating, but a bit more unclear what effect youth involvement had on policy level (10).

Youth participation with the purpose of advancing health equity takes on different approaches in how young people are recruited, how power is shared between young people and adults, and in which phases the young people participate (16). In Swedish mental health policy, however, young people do not seem to be understood as experts with relevant knowledge for youth mental health promotion (17). Furthermore, involving young people with different backgrounds, to not risk reinforcing existing inequities, seems to be a struggle in practice (10). Therefore, strategies must be developed to involve young people being marginalized, and not only involve more privileged young people (16). Despite well intentions and knowledge of inequities in youth participation, there seems to be a lack of studies focusing on the role of practitioners in youth participatory initiatives, how practitioners developing health promotion could alter their practices to equitably involve young people with different backgrounds and experiences. Thus, this paper aims to (1) describe how participatory action research was used to develop a model for practitioners to equitably involve young people in mental health promotion initiatives and (2) present the finalized model, Steps Toward Justice.

## 2 Methods and materials

### 2.1 Study design

This study was conducted as a participatory action research (PAR) project. It adopted a critical PAR approach inspired by Kemmis et al. (18), as the project largely aimed to change the social practices of practitioners, by practitioners themselves (18). Kemmis et al. (18) defines practices as being composed of sayings, doings, and relatings. The practices are made possible by practice architectures in which cultural-discursive, material-economic, and social-political arrangements form the conditions for practices. In the current study, the act of involving young people in mental health promotion is considered a practice. This practice does not only happen between the practitioner and the young person, but is also shaped by its cultural-discursive, material-economic, and social-political arrangements and conditions. Changing a practice requires more than just enhancing knowledge about it; its arrangements and conditions must also change, challenging the practice architecture (18).

This study was an outcome of a larger project aiming to take a systems-based approach to youth mental health promotion in the county of Blekinge in the south of Sweden (19). A group of approximately 80 practitioners working with young people in different sectors and approximately 50 young people aged 13–16 from different schools partook in the project. A smaller group of practitioners interested in youth participation was formed as part of the larger project. The smaller group formed a PAR team and met seven times during the spring of 2024, both together with the larger project and separately. The PAR team composition varied in numbers between four and eight as some practitioners had to leave the project due to time constraints, and new practitioners joined. The PAR team held a diverse occupations, including school principal, youth leader (third author), regional development officer focusing on children and young people (fourth author), municipal officer focusing on schools, and a PhD-student with experience of working with health promotion and young people (first author). The research was conducted in three steps described below.

### 2.2 First step: constructing a model

Through previous experiences and knowledge, the PAR team identified the need to focus on the equal and fair involvement of young people in these types of mental health promotion efforts. A model for equitable involvement was developed through system-mapping of the obstacles and possibilities for involvement of different groups of young people (20). It was used as a foundation for discussions, amongst the PAR team and with other practitioners and young people participating in the larger project. The idea was that the model could be used to critically reflect about equal and fair involvement of young people in the planning and conducting of mental health promotion initiatives. Moreover, it was intended to accommodate the bureaucratic reality with time and budget constraints, as well as the legal requirements, many of the practitioners found themselves in. Throughout the study, a differentiation was made between youth involvement and youth participation. The model focuses on involvement, because it is aimed at practitioners who plan the mental health promotion efforts, as they often have the power to choose which young people to involve and to create spaces for youth participation. The first version of the model consisted of five steps of actions to be taken to, in a more fair and equal way, involve young people with different experiences and backgrounds in mental health promotion. The model was meant to be read from the bottom up.

### 2.3 Second step: developing the model

The initial model constructed by the PAR team was further developed through focus group discussions with a total of 12 young people (Table 1) and 10 practitioners working with and for young people (Table 2). In total six single focus groups were conducted in this part of the study, three with young people and three with practitioners. Focus groups were chosen as it is a good method to collectively discuss an issue and develop a product or a program (21). This was seen as important in developing the usefulness of the model for practitioners, but also the relevance for young people. Four people in total (first, third, and fourth author, and a fourth person working as a youth leader) moderated the six focus groups, with each focus group having two moderators. The focus group discussions were recorded using audio recording. Separate discussion guides were used for young people and for practitioners, which are available in the Supplementary material. The five steps of the model were printed and cut, making it possible for the participants to move and discuss the preferrable order of the steps. The focus groups started with questions about involvement and equity in general and then moved on to discussing the model. The intention from the PAR-team was that the steps should be read from the bottom and up, it was, however, read in different ways by the participants in the discussions.

**Table 1:** Focus groups with young people.

Focus group	Gender	Age
A	Female	16
A	Female	18
A	Female	17
A	Female	16
B	Female	13
B	Female	13
B	Male	14
B	Male	14
C	Female	12
C	Female	13
C	Male	15
C	Male	15

**Table 2:** Focus groups with practitioners.

Focus group	Gender	Profession
D	Female	Project manager for civil society
D	Female	Teacher
E	Female	Project manager for civil society
E	Male	Social pedagogue
F	Female	Social worker
F	Female	Social worker
F	Female	Social work coordinator
F	Male	Social worker
F	Male	Youth leader
F	Female	Youth leader

#### 2.3.1 The young people

The young people who participated in the focus groups were aged 12–18 years old and none of them had participated in the previous step of the study. They were recruited from youth centers in a municipality in Blekinge; the chosen youth centers were in different areas of the municipality in an effort to include young people with different backgrounds. Moreover, the three focus groups were conducted at the youth centers, which the participants often visited and were familiar with. One of the moderators of each focus group was a youth leader who knew the participants. The young people did not receive the model before the focus group to limit the workload needed for participation.

#### 2.3.2 The practitioners

**Figure 1:**
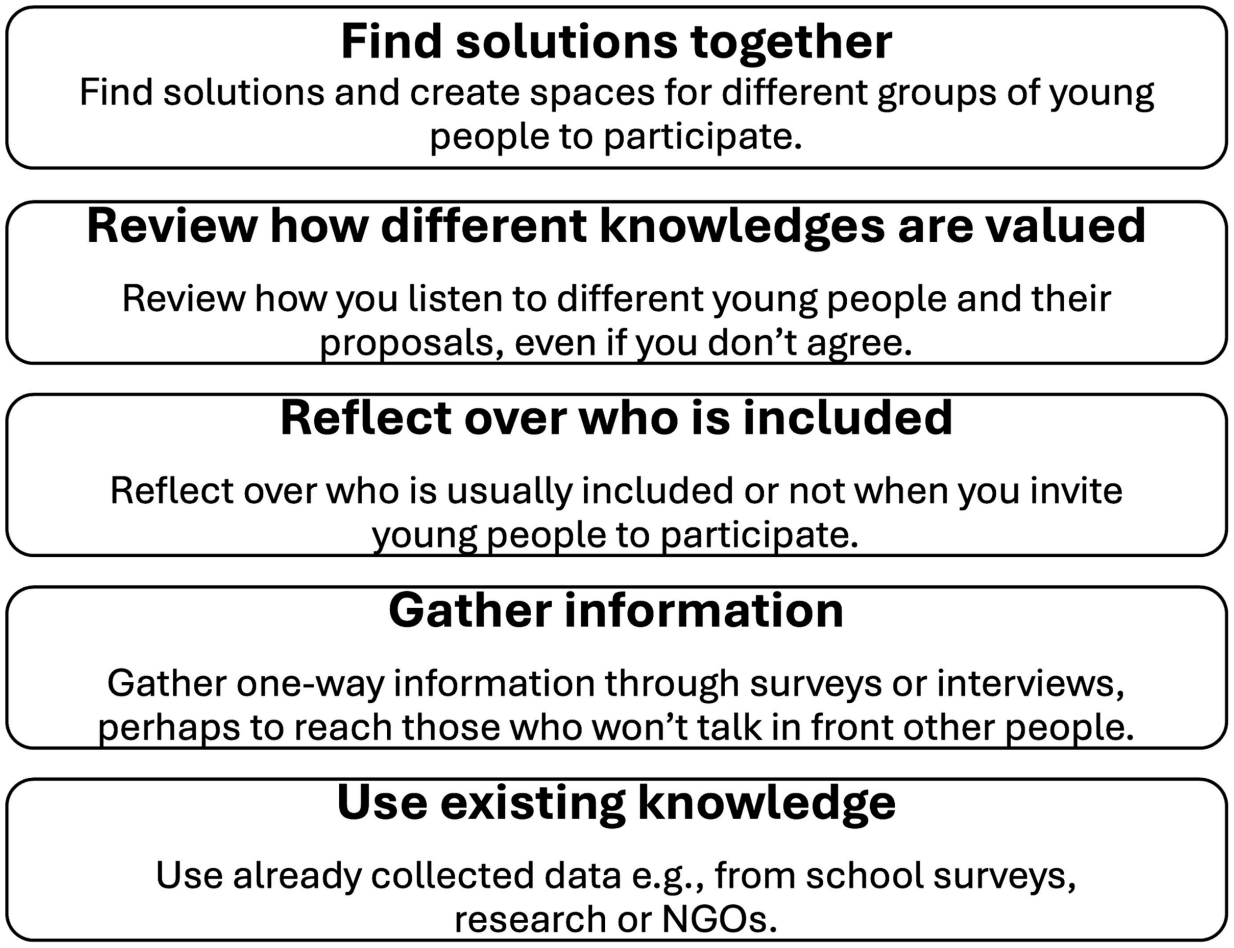
The first version of the model, which was used as a base for discussion in the focus groups.

The practitioners who participated in the focus groups worked in the county of Blekinge with young people directly or with issues regarding young people and had an interest in discussing equity and youth involvement (see Table 2). They were recruited through professional contacts of the PAR team as well as from the larger project from which the study was formed. Three of the practitioners who participated in the focus groups were part of the larger project from which the study originated, and seven were not. The practitioners received the model a week before the focus groups were conducted, with the instructions to look it over and possibly discuss it with some colleagues. All of the participants had looked at the model beforehand and some had discussed it with colleagues to encourage deeper reflections going into the focus group (Figure 1).

### 2.4 Third step: finalizing the model

The audio recordings of the focus group discussions were transcribed and the transcripts were used in the analysis. The analysis was inspired by the Krueger and Casey (21) approach to analyzing focus group discussions. First, the transcripts were, printed on paper and cut into sections containing similar content, by the first author. The focus groups with the young people and with participants were marked with different colors but analyzed together. Three members of the PAR team (first, third, and fourth author) and two senior researchers (second and seventh author) took part in the analysis and gathered in a big room. The sections were sorted and, through discussions, re-sorted into one of the five steps of the initial model or into new steps formed throughout the analytical process. Both the words used and the interpreted meaning behind those words were considered in the joint discussion of similarities and differences of the sections. If the group had different interpretations, they were discussed until an agreement was reached. Thus, through the analysis process, three steps were added to the original five steps. The resulting eight steps were divided into three phases of equitable involvement, with each step being conceptualized as a call-to-action.

## 3 Results

The study resulted in the model Steps Toward Justice—a model for the equitable involvement of young people in mental health promotion. The model consists of three phases: preparing, conducting, and applying. Each phase contains steps of actions to be taken toward a more just and equitable youth involvement in a mental health promoting initiative. The three phases indicate which actions can be taken in each phase of youth involvement in a mental health promoting initiative.

**Figure 2:**
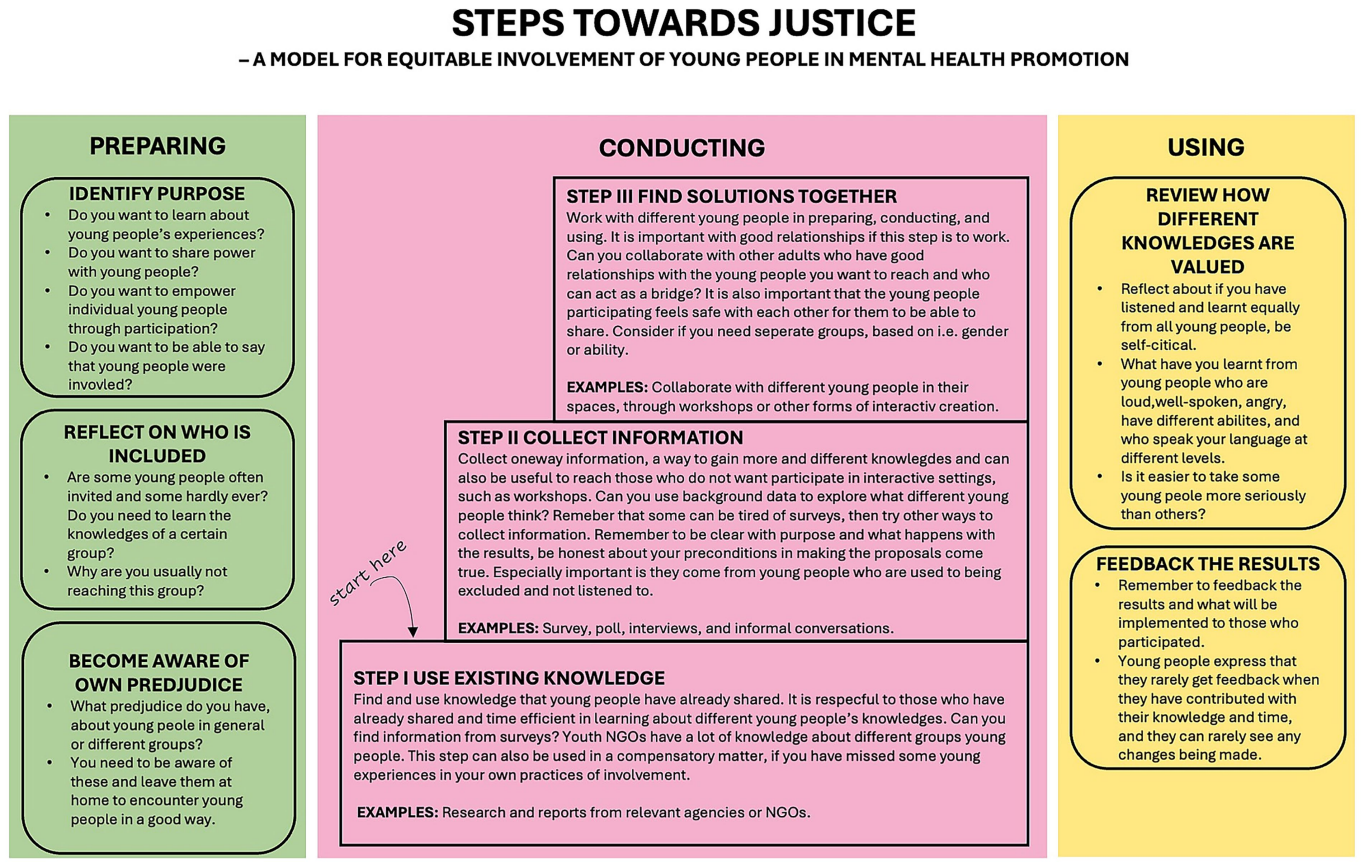
The final model Steps Toward Justice.

In this section, the model is presented first (see Figure 2) and then the analysis from the focus group discussions is presented to provide further depth and practical examples of the steps in model, with quotations from practitioners and young people. The quotations in the model (presented in white speech bubbles) aim to illustrate the examples and suggestions from the young participants.

### 3.1 Phase I: preparing

The preparing phase included what the participants saw as necessary for practitioners to consider before starting to involve young people in the process. It involved self-reflecting on the purpose and motives of involvement, on who is usually included and excluded, and the practitioner’s own preconceived ideas.

#### 3.1.1 Identify purpose

The importance of having a clear purpose was emphasized by both the young people and practitioners. The purpose of involving young people in mental health promoting projects and processes could be both to benefit the project, by receiving input from young people, and to benefit the individual young person by giving them possibility to grow and gain new experiences. Both the practitioners and young people stressed the importance of having a clear purpose for involving young people. The practitioners argued that the Convention on the Rights of the Child requires them to involve young people, but that it can be challenging to include everyone and have an equitable involvement. They also considered young people to be experts on the life and needs of young people.

Depending on the purpose, different approaches could be taken. If the purpose is to benefit the participating individual, the participants emphasized the importance of inviting everyone to participate. On the other hand, if the purpose is to benefit the work of a project or an organization it could be more important to find a representative sample of participants, as discussed by both practitioners and young people. The young people discussed the issue of purpose and the timing of involvement. They shared the frustration of being involved late in the process, when the decisions had already been made.

“Sometimes it feels like the survey is just for show, that there is no consideration of the answers; the decision has already been made. But to be able to say, ‘we let young people be included’, you do the survey.” (Young person 1).

They interpreted this as a form of tokenism, where young people were involved merely so that a project could say that they had involved them, rather than caring about young people’s expertise.

#### 3.1.2 Reflect on who is included

The young people and practitioners alike stressed the importance of reflecting on who is invited to participate. In their experience, those invited to participate are often young people who are already involved in school, in organizations, or politics. Sometimes groups of young people, such as a school class, can take a vote to elect representatives, but this can also be unfair as those who are popular or fit into the norm are more likely to be elected, the young participants explained. They considered it important to identify who is and is not usually included, in order to work differently going forward. Fair and equitable involvement is the responsibility of everyone involved in a project and it is should be considered at every step of the process.

“What is problematic in this case is when we get an assignment from a municipality to come and get the opinions of children and we get the selected group from the school ‘these are the children you should talk to’… And we can ask, ‘on which grounds were these children chosen to be part of the focus groups?’ ‘It is because these children usually talk’. And then the problem is, I cannot say that this is representative for all children.” (Practitioner 1).

The young participants described experiences of being excluded from participating in specific events and a general feeling of not always being listened to by adults, for instance, by teachers in the classroom. They referred to this as unjust, often seeing that other young people are more listened to or invited to participate. The practitioners were aware that not all young people are equitably invited to participate, but they also explained that it is more challenging to involve those who are quieter or who do not usually participate in activities outside of school. It is not enough to say that everyone is welcome; this was the experience of both practitioners and young participants. The practitioners also mentioned that they have occasionally been able to include those who often do not feel listened to and that this inclusion has led to concrete changes.

Both the young participants and practitioners discussed different knowledges. What is most important is not always getting everyone involved but identifying which young people have the specific knowledge needed in this project. The young participants emphasized this as a way of finding participants who have a passion or interest in the issue, not just those who want to play around.

#### 3.1.3 Become aware of own prejudice

In order to equitably involve and listen to young people, adults need to be aware of their own prejudice. The young participants especially emphasized the need to be aware of one’s own racist, sexist, or heteronormative ideologies and prejudices and to not bring these when meeting young people.

You should not discriminate and more like… ‘Here I am with open arms as you are’, and not ‘since you are this, I will act like that’. But welcome everyone in the same way and really try to not treat individuals differently based on culture or background. (Young person 2).

Adults aiming to involve young people should also be aware of prejudice and power imbalance between the young people participating. Having knowledge about the norms and expectations in the specific group of young people could help practitioners create safe spaces where everyone feels comfortable expressing themselves.

### 3.2 Phase II: conducting

The conducting phase includes what the participants found important in the practical phase of involving young people. It includes continuous reflection about similar issues to the preparation phase and about the practicalities of involving young people equitably, such as making use of what young people have already contributed with, finding more simplified ways of collecting information from young people, and figuring out how to collaborate with young people to find solutions. The steps in this phase can be used as a staircase; for instance, if resources are limited, practitioners could choose to only take Step I in a particular project instead of involving the group of young people who are “always” included.

#### 3.2.1 Step I use existing knowledge

Both groups of participants pointed out that young people are frequently asked to fill out surveys in school, contributing to research and reports. Reading previous research and reports can never be equally important as talking to young people directly, the young participants expressed. The practitioners on the other hand discussed that making use of already collected information could be a time-efficient way to find information, as time and budget constraints are part of the everyday work life of the practitioners. They raised that it is important to consider which young people and in which settings the studies were conducted, to evaluate their transferability to a specific project.

Many of the [mental health promotion programs and materials] that are available, they are not adapted to rural areas. If you want to find inspiration from other places, then it’s from a different context. Our children have other problems and everyday challenges. So, I also think about that, from an equity perspective. (Practitioner 2).

Although the practitioners raised the limitations of only relying on existing knowledge, they also emphasized the importance of examining the surveys and other forms of data collection that young people in a particular project have already participated in. Using existing knowledge could give a more varied opinion and experience than only asking a few young people directly. It is also a way of honoring previous contributions and showing young people that their participation can make a change.

#### 3.2.2 Step II collect information

Different purposes and different groups of young people could require different ways of collecting information. The practitioners argued that talking to young people gave richer answers than surveys and that this process could also help build relationships. The young participants expressed frustration with the number of surveys they were required to fill out. They often found them too long, and they rarely got information about what happens with the result of the surveys, which made them not take the surveys seriously. Nevertheless, some of them believed surveys could still be useful if used in the right way.

I think it’s okay with surveys if you take the answers into account and try to make improvements. Rather than just doing the survey and getting some statistics. ‘Oh it’s like this’ and then just blow it off. (Young person 3).

Surveys could also be useful, the young participants expressed, as they are anonymous and could make it possible for people who are unsafe to express themselves in front of other young people. Similarly, the young people expressed the need for safe spaces if everyone is going to feel safe to share their opinions in person. One way of doing this could be having separate groups, for example, of LGBTQI+-people, as one of the participants identifying as LGBTQI+ expressed.

The practitioners reported that they often conducted focus groups or workshops when they wanted to involve young people, and while this could be a great method, it could also exclude those who do not feel safe or comfortable in these types of group settings. Both the practitioners and young participants underscored the importance of informal conversations: People who do not usually want to participate in projects and express their views in front of other people still have important knowledge to share, and one way to involve them is to have informal chats in the school corridors or while doing activities such as crafts. Journaling could also be a way for young people to share experiences and opinions if they are more comfortable with writing than talking.

Some of the practitioners worked directly with young people on an everyday basis, whereas others worked strategically and only met young people occasionally. Since both groups recognized that trust was essential to involving young people, they suggested that those working strategically could collaborate with practitioners who worked closely with young people and had already built trust. This could be particularly useful in reaching young people who lack trust in adults and who are not usually invited to participate. They also suggested combining different methods of collecting information if the project’s time and budget allow this.

#### 3.2.3 Step III find solutions together

For both the practitioners and young participants, the ideal way to involve different young people in mental health promotion was to find solutions to mental health issues and ensure equitable involvement together. However, the practitioners considered this the most difficult and demanding of resources, whereas the young participants gave suggestions on how to achieve it.

You do not always have to get everyone’s opinion. But you have to get a varied opinion. (Young person 4).

The practitioners experienced that a more participatory approach of working with young people led to higher engagement and achieved results that are more in line with young people’s experiences.

In order to reach the stage of finding solutions together, practitioners and young people require trust, good communication, and individual solutions. The young participants stressed that young people need to feel trust toward the practitioners working together with them and to be well-treated by them, describing previous experiences of practitioners not treating them well by being disrespectful, yelling at them, expressing prejudice, or only pretending to listen. The individual solutions suggested by the practitioners include having a mix of group and individual participation and meeting individuals in spaces that feel safe for them. Moreover, fostering a personal relationship may contribute to building trust. The young participants made this suggestion in connection with the issue of having negative experiences with adults or little experience of being taken seriously. They proposed that practitioners who do not know the young people they are trying to involve beforehand could collaborate with practitioners who do, to build trust and reach young people with previous negative experiences of adults or exclusion.

None of the young people in the focus groups shared experiences of being involved in a project at this level of involvement. They stressed the importance of being listened to and included; in addition, they pointed out that sometimes it is better for practitioners to take some responsibility rather than place it all on young people, since practitioners have more time for administration than young people in general.

### 3.3 Phase III: applying

The application phase contains what the focus-group participants thought was important for practitioners to consider at the end of a project. This phase still revolves around practitioners’ self-reflection and critical thinking about how the different experiences and knowledges of different young people should be interpreted and utilized, but it also concerns how the results of the project are reviewed and how feedback is provided to the young people who contributed with their time and expertise.

#### 3.3.1 Review how different knowledges are valued

By reviewing how different knowledges were valued, practitioners referred both to the knowledge of young people in general, as opposed to adult knowledge, and to the knowledge of specific groups of young people. The practitioners’ awareness of their own prejudice and assumptions becomes relevant again in the utilization of knowledges gained through the process. That is, practitioners should take the experiences and opinions of young people into consideration regardless of whether they agree with them. This point was also raised by the young participants, who emphasized that valuing young people’s knowledge requires practitioners to set aside their own plans and be willing to adopt young people’s suggestions.

Because they always say ‘yes, let us do that’ and then they do nothing. And they do the thing that they want to do. (Young person 5).

The practitioners also stressed the need to review what the knowledge from individual young people says about their environment, for instance, what the experiences of a young person at a school reveals about that school. This also involves considering different experiences of the same environment. Even if a diverse group of young people is involved in a health promotion project, the project’s practitioners have to pay attention to how they listen to different young people and be aware of potential bias influencing whose suggestions they see as valid and whose suggestions they disregard.

#### 3.3.2 Feedback the results

The practice of providing feedback on the results of a project to the young people who were involved was rare, according to the young participants. They expressed frustration at never getting to know what happened to the survey results or why their proposals were not implemented. Even if proposals are not implemented, they would like to know why and not just hear a “no.” The young participants underscored this issue in particular, stating that not getting feedback in the past has led to them not taking surveys or other forms of involvement seriously.

Well, you are not going to answer truthfully as long as you know that nothing is being done. (Young person 4).

Different ideas on giving feedback were suggested by the practitioner participants and young participants. For instance, it could involve going over the results of a survey on group level and discussing what they mean or showing concrete actions or changes that have been made due to the participation of young people. The young participants expressed both understanding and being frustrated at budget constraints and the fact that bureaucratic processes take a long time. They were also frustrated by the fact that when young people who are part of a minority offer a different suggestion than the majority, their voice will be overpowered by the majority.

## 4 Discussion

This study aimed to describe how a model for practitioners to equitably involve young people in mental health promotion initiatives was developed through participatory action research and to present the model. Accordingly, this section comprises two parts: the first part discusses the methodological aspects of developing a model for equitable involvement through PAR, and the second part discusses the focus-group outcomes and the resulting model, Steps Toward Justice.

### 4.1 Methodological discussion

Employing the critical PAR approach to develop a model for health promotion practices benefited the study in several ways. First, practitioners who work in everyday settings where mental health promotion takes place have expertise in these practices. Practitioners with such expertise were involved in every step of the study, from identifying the problem to designing, conducting, and analyzing the results to finalizing the model. Second, people of different ages and professional backgrounds contributed to the model in different ways, which strengthens the legitimacy of the study (18). However, recruiting participants for the practitioner focus groups was challenging, and some participants dropped out on the day of the interview, leaving two of the focus groups with only two participants, while the third focus group had six participants. Having larger focus groups overall could have contributed to more varied experiences and opinions in the discussion. Further, the fact that most of the participants were female could be considered a weakness of the study and some perspectives from other genders might have been missed. Nevertheless, the limited number was to some extent compensated by all the participants in the focus groups being experts on the topic (see 21), which resulted in engaged discussions around the model. In line with the PAR approach of the study, the focus group moderators were also practitioners (similar to the participants), which further deepened the discussions and contributed to building trust between the participants and the moderators (18).

Two senior researchers (second and seventh author) conducted the analysis together with the PAR team (HG, MP, and HSA) and two additional senior researchers (fifth and sixth author) participated in the writing of analysis and construction of the final model. All of the authors participated in planning how the focus groups were to be conducted. This further strengthened the analysis as knowledge of research and of practice were combined, but also as the senior researchers, who had not conducted the focus groups or been involved in developing the first draft of the model, could provide an outside perspective. Member-checking was conducted, as the participants in the focus groups were invited to review the final model before submission which contributes toward the credibility of the study and the usefulness of the model (22). Only minor language corrections were made to the model as a result of the member-checking. The model was also presented at a workshop for practitioners interested in the equitable involvement of young people in health promotion, creating an opportunity to receive feedback on the model’s comprehensibility. The feedback from the practitioners indicated that the model was understood as intended and no further changes were made.

The young people involved in the focus groups raised the issue of adults consulting young people and then not taking any action or reporting back on what happened with the knowledge they shared. This could be understood as research fatigue, which particularly can be a problem for individuals belong to groups being marginalized as they are often invited to participate in research (23), even though they do not always end up participating in practice. This could also be considered an ethical problematic aspect of this study, especially as some of the young participants had experiences of being marginalized and not listened to (see 24). One of the PAR team members works at the youth centers where the young participants were recruited, so they will hopefully be able to continue the dialogue and more easily report back the results of the study to these participants.

Conducting a study about the equitable involvement of young people requires self-reflection and criticism of equity in relation to involvement in this study. Although the study as a whole had a PAR approach, it was PAR with practitioners, and the young people contributed as traditional participants in qualitative research. The young people who participated in the larger project from which this study originated could not be part of the PAR team due to limited time, they instead acted as consultants for the PAR team instead (18). Furthermore, the purpose of this study was not to implement the ideas of young people, but to develop a model for practices. Namely, the study focused on the practices of practitioners, both to highlight the practitioners’ responsibility for equitable involvement, and to gain their knowledge of their everyday work situation in which mental health promotion practices take place. Discussing both the research issue and the model with practitioners from different professions, in different geographical locations, and in different phases of the project contributed to developing a model that has the potential to be transferable to other settings working with young people as well (25).

### 4.2 Discussion of the results

The Steps Toward Justice model was a result both of the work of the PAR-team and the practitioners and young people in a large mental health promotion project as well as of the analysis of the focus groups with additional practitioners and young people. Kemmis et al. (18) way of describing practices as sayings, doings and relatings, connects to how the focus group participants discuss the practices of each step in the model. For example, not enough to mean well or to say that everyone is welcome, it also needs to be reflected in the doings and in the relatings to become a practice of equitable involvement. Further, the practitioners, in the focus groups, in the PAR-team as well as in the larger group from which the study originated, further provided knowledge of a bureaucratic reality where the most ideal strategies for equity and participation were not always feasible. This knowledge further, together with that of the young people, contributed toward making the model more concrete and precise in its suggested actions.

One of the main topics discussed by both young people and practitioners was the importance of relationships and the role of informal conversations in inviting and engaging young people to participate, especially young people who are used to feeling excluded and experiencing marginalization. Similar experiences were reported in a participatory project by Young et al. (26), which emphasized relationship building is essential for youth participation and especially to create equitable and inclusive participation. Personal relationships are also an important part of practices and the theory of practice architecture. Practices take place in different spaces where practitioners and young people meet, in language, in activity and work, and in relationships of solidarity and power in the social space (27). Therefore, it is important for practitioners to reflect on their own prejudice and on what they bring to the social space of practices during interactions with young people. They should also recognize the role of building relationships with young people, for instance, through informal interactions. The knowledge gained from informal conversations can be crucial for equitable practices, especially for involving young people who are usually not invited or do not usually participate. It can also be useful, although less used, in qualitative research (28). In this study, informal conversations were also important for the development of the model, with both practitioners and young people, in the first step of the study.

Although the focus of the model is the practices of practitioners, the involvement of young people to share their expertise was crucial for developing the model. The young people were critical of the “everyone is welcome” approach, which could be seen as part of the more general, and vague, mental health equity discourse (17). The young people in general expressed the need to be clear and transparent, with purpose and with what is actually possible to change. This aligns with the findings from Flodgren et al. (10) where young people who had participated in public health initiatives expressed positive experiences of being able to express themselves, but some were also disappointed as their views had not been taken into account. In this study, the young participants’ contribution to the model, through their examples of equitable and inequitable practices of involvement, was especially important for making the model concrete and practical.

The practitioners emphasized the importance of being self-critical and transparent, for instance, when identifying which young people are usually included and who are not; both in broader sense through systemic inequities, similar to Ozer et al. (16), and in the specific context, for instance, at a certain school or youth center. When the practitioners discussed the importance of equitable involvement it was mainly from a children’s rights argument (8), perhaps unsurprising given the increased focus on the Convention on the Rights of the Child in a Swedish public sector context (15). However, some participants also raised the importance of equitable involvement to enable mental health equity on a more structural level.

The Steps Toward Justice model can be useful for practitioners wanting to involve young people when planning and conducing mental health promotion, for instance, at schools or youth centers. It can initiate a process of reflection over equitable practices and enable practitioners to take Steps Toward Justice through concrete actions of involvement, moving beyond a vague discourse of “everyone is welcome.” The purpose of the model is not to describe everything that could be done to involve young people equitably but to gather and synthesize the different knowledges of health promotion practices and equity developed through this PAR study.

## 5 Conclusion

The PAR approach by practitioners working with young people contributed toward developing a model that considers inequities and injustices while simultaneously being viable for use by practitioners working in non-optimal environments with budgets and time constraints. The PAR approach and the involvement of both practitioners and young people in the study illustrate how practitioners and young people can work together to create a model. The participants contributed toward creating a concrete model with practical examples. The young participants mainly contributed with experiences of being or not being invited to participate and with their emphasis on the importance of being critical about their own prejudice and values as practitioners wanting to practice equitable involvement. The practitioners primarily contributed with their knowledge of bureaucratic reality and by making the model practically useful. In conclusion, the Steps Toward Justice model was developed for practitioners wanting to take some Steps Toward Justice through pursuing equitable involvement in youth mental health promotion, and possibly other initiatives with young people. Future studies could further evaluate and develop the model for practical application.

## Data Availability

The raw data supporting the conclusions of this article will be made available by the authors, without undue reservation.
