# Supplementary material for: Steps Toward Justice: a model for equitable involvement of young people in mental health promotion
Source: Front Public Health. 2025 Nov 13;13:1636799. doi: 10.3389/fpubh.2025.1636799 (PMC12657437; doi:10.3389/fpubh.2025.1636799)
Supplement: Supplementary file 1 [file Table_1.docx]

**Guide for focus group discussions.**

**General questions**

1. Have you worked with involvement and participation of young people? In what way?
2. If you think back, how are young people usually recruited and involved when you want to learn about young people’s experiences?
3. Can you reflect on who is included and who is not?

**Questions about the model**

1. What do you think of the proposals in the model? Do you have other suggestions? If you were to put the proposals in order, which one would you start with?
2. Can you reflect on if and how this type of model could be useful in your work?
3. From our discussions today, what is the most important for us to consider?
4. Is there anything we forgot to ask that you would like to share?

**Guide for focus group interviews with young people**

**General questions**

1. Have you been part of a focus group or interview before? If so, what was it about?
2. Does the school, youth center, or organizations that you are a part of ask about your opinions and experiences? How have they asked?
3. When have you been able to share your opinions in schools, youth centers or organizations? Did you find out what happened afterwards?
4. Are some young people often involved and get listened to, and are some rarely getting invited?
5. Does it matter if some get invited and some do not? In what way?

**Questions about the model**

1. When we have talked to young people and to adults who work with young people, we have heard that adults in general are bad at asking what young people think and want. We want to make it easier for adults who work with young people to ask and learn about different young people’s experiences. We have five proposals on how this could be done. What do you think of these proposals? Do you have other suggestions?
2. If you look at the proposals, which one should they start with? Put them in the order that you think would be best.
3. From our discussions today, what is the most important for us to consider?
4. Is there anything we forgot to ask that you would like to share?
